# Supplementary figures and images for: Covariation between microeukaryotes and bacteria associated with Planorbidae snails
Source: PeerJ. 2023 Dec 19;11:e16639. doi: 10.7717/peerj.16639 (PMC10740603; doi:10.7717/peerj.16639)

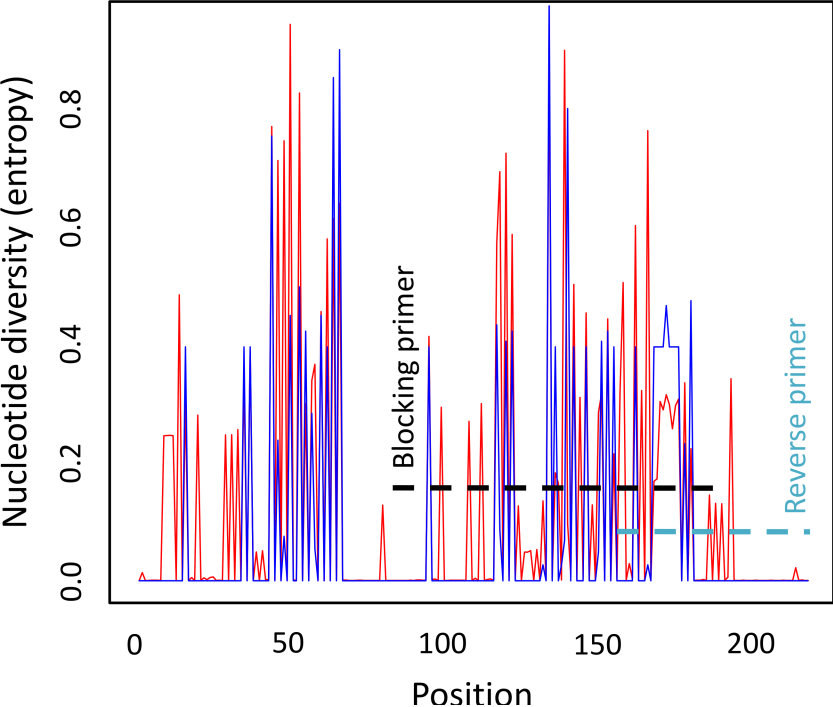

Supplement: Supplemental Information 1 — Blue and red lines correspond to Heterobranchia and metazoa-free sequences, respectively. Dashed lines indicate positions of reverse (in light blue) and blocking (in black) primers. [file peerj-11-16639-s001.pdf]

Species richness

Microeukaryotes

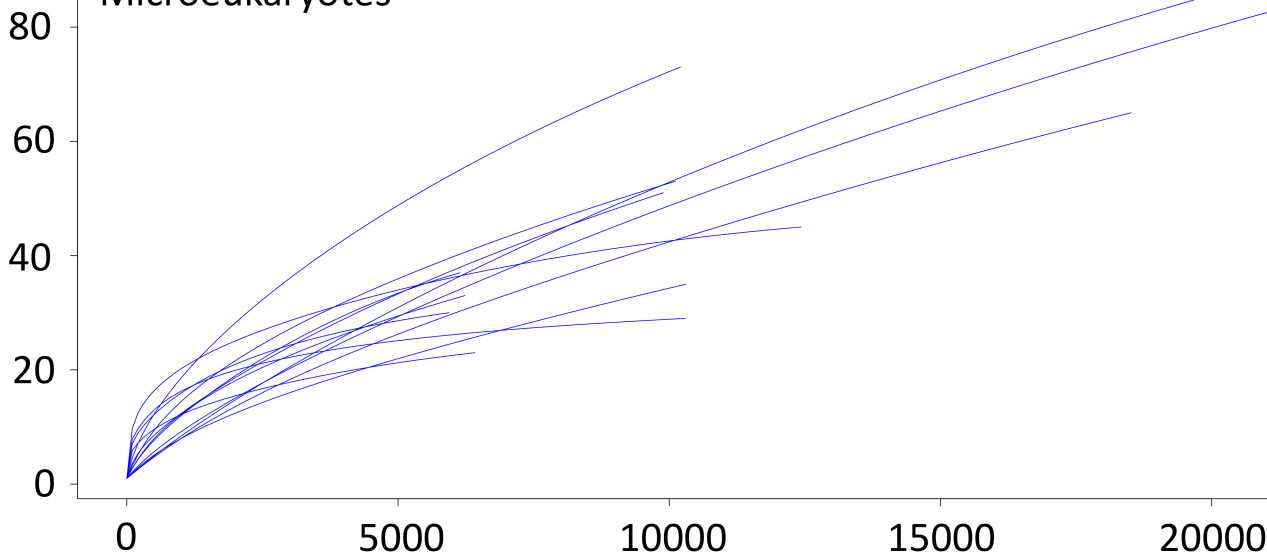

Bacteria

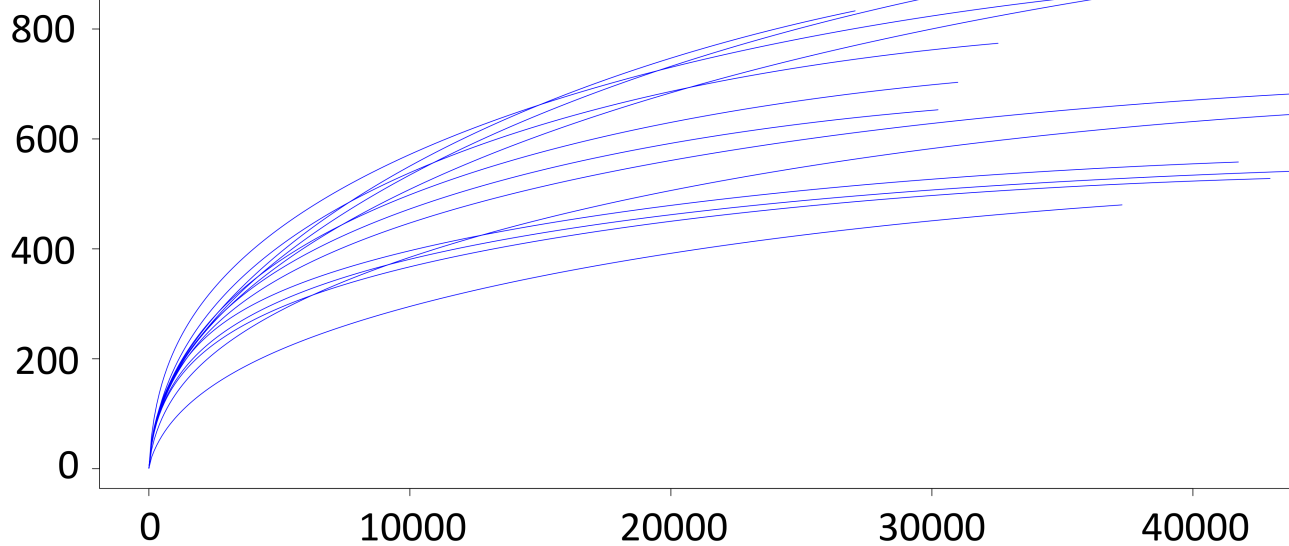

Number of sequences

Supplement: Supplemental Information 2 [file peerj-11-16639-s002.pdf]

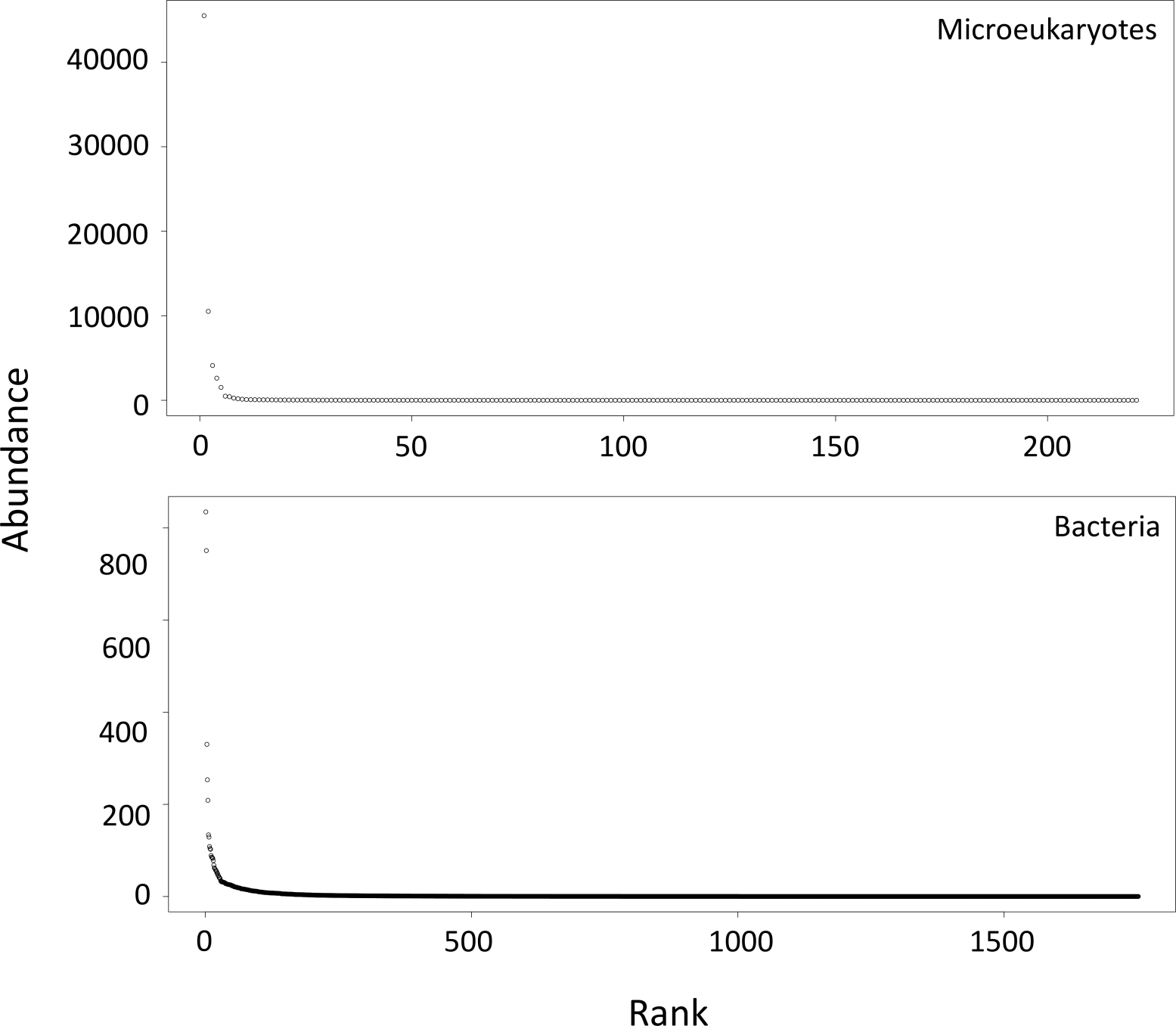

Supplement: Supplemental Information 3 [file peerj-11-16639-s003.pdf]
